# CYP27B1 Enzyme in Psoriasis: A Preliminary Study of Immunohistochemical Observations

**DOI:** 10.3390/life14010015

**Published:** 2023-12-21

**Authors:** Iulia-Alexandra Paliu, Maria-Victoria Olinca, Simona-Laura Ianosi, Claudia-Valentina Georgescu, Adina Turcu-Stiolica, Magdalena Diaconu, Cristiana-Iulia Dumitrescu, Andrei-Adrian Tica

**Affiliations:** 1 Department of Pharmacology, University of Medicine and Pharmacy of Craiova, 200349 Craiova, Romania; iulia_paliu@yahoo.com (I.-A.P.); diaconumagda@yahoo.com (M.D.); dumitrescu.cristiana@gmail.com (C.-I.D.); ticaandrei2002@yahoo.com (A.-A.T.); 2 Department of Pathology, “Carol Davila” University of Medicine and Pharmacy, 020021 Bucharest, Romania; 3 Department of Dermatology, University of Medicine and Pharmacy of Craiova, 200349 Craiova, Romania; 4 Department of Pathology, Clinical Emergency County Hospital of Craiova, 200349 Craiova, Romania; cgeorgescu2001@yahoo.com; 5 Department of Pharmacoeconomics, University of Medicine and Pharmacy of Craiova, 200349 Craiova, Romania; adina.turcu@umfcv.ro

**Keywords:** 1-α-hydroxylase, CYP27B1 enzyme, vitamin D, psoriasis

## Abstract

Connections between vitamin D and psoriasis have been a matter of interest for the past decades, with its active metabolite, 1,25(OH)_2_ vitamin D, being valued for antiproliferative and immunomodulatory effects. However, none of vitamin D’s actions could be possible without the CYP27B1 enzyme that bio-activates this metabolite of interest. In order to see if there is any link between the enzyme expression and the disease’s particularities, we conducted a preliminary study that involved 11 skin biopsies of patients with mild (n = 4) or moderate to severe psoriasis (n = 7). The cell proliferation antigen Ki67 and the CD45RO+ marker were also assessed. Compared with healthy skin, in psoriasis, it is reported that the enzyme’s expression seems to be more ubiquitous, but a clear correlation between the disease’s severity and the CYP27B1 expression was, to our knowledge, lacking. We found that, in patients with very mild psoriasis, the enzyme expression was observed in the epidermal stratum basale in a similar manner as in healthy skin specimens. Contrary, for higher severity scores, a divergent result was observed, with the enzyme being either variably spread in the epidermal stratum spinosum or completely absent. Unlike malignant diseases, a significant connection between CYP27B1 and Ki67 (*p* = 0.313) or CYP27B1 and CD45RO+ (*p* = 0.657) does not seem to be relevant in psoriasis.

## 1. Introduction

Psoriasis is a chronic disease of the skin that involves dysregulation of the immune system’s function, with an exacerbated inflammatory state mediated primarily by the T helper cells type 17. This fact will be followed by excessive cytokine production and an exaggerated proliferation of the keratinocytes [1]. The disease’s etiology remains unclear, but it is generally accepted that a yet unknown antigen stimulates the migration of the memory-effector T cells into the skin, most of which can be identified as CD45RO+ memory-effector T cells [2].

It was demonstrated that 1,25(OH)_2_ vitamin D, the active form of vitamin D, has a significant regulatory action on the abnormal proliferation of keratinocytes. On an immunological level, this metabolite reduces the proliferation of the T-cells by inhibiting the secretion of pro-inflammatory cytokines such as interleukin (IL)-2, IL-6, IL-8, and interferon (IFN)-γ and by promoting the activity of the anti-inflammatory cytokine IL-10 [3]. Additionally, recent studies indicate 1,25(OH)_2_ vitamin D as a protective factor for mitochondria of some cells, including skin cells, as it seems to be involved in the modulation of oxidative stress levels and, therefore, in the mechanism of inflammation induced by Reactive Oxygen Species (ROS) [4].

Furthermore, an interesting report indicated 1,25(OH)_2_ vitamin D as an inductor of IL-33′ expression and its receptor ST2, a cytokine suggested to act in both pro-inflammatory and anti-inflammatory ways in psoriasis [5].

Considering all mentioned above, vitamin D appears to be a paradigm for future promising therapies in psoriasis. Vitamin D derivatives, calcitriol, calcipotriol, maxacalcitol, tacalcitol, and hexafluroro-1,25(OH)D, were proved to be effective in treating this disease by numerous clinical trials [6,7]. Among them, the most utilized pharmaceutical form uses calcipotriol in association with a glucocorticoid, and it can be used either as monotherapy or as an add-on to biological or non-biological therapy even in moderate to severe cases of psoriasis [8].

Vitamin D is not an active compound per se and it needs to be metabolized in order to be activated. The first place of bio-activation is localized in the liver [9], where CYP2R1 is the main enzyme involved in the conversion of vitamin D into the 25-OH vitamin D metabolite [10]. Consequently, in order to interact with the specific Vitamin D Receptor (VDR), 25-OH vitamin D needs to be converted into the physiologically active metabolite 1,25(OH)_2_ vitamin D, a process mediated by CYP27B1 enzyme [11,12,13].

CYP27B1, also named 1-α-hydroxylase, is an enzyme first discovered in the kidney, more precisely in the proximal tubule, in the early 1970s, and it was considered for over a decade the only source of bio-activation for the active metabolite of vitamin D, 1,25(OH)_2_ vitamin D [11,14]. Its membership in the Cytochrome P450 (CYP450) family was suggested by the sequence of the amino acids and the similarity with the rat CYP27A1 enzyme [15], and judging by the biochemical analysis, we can add that the enzyme, besides the cytochrome component, it is also a mixed-function oxidase [16].

Although the main localization of the enzyme is in the renal cells, the circulating levels of 1,25(OH)_2_ vitamin D would be too low (approximately 10^−11^ mol/L) to achieve a therapeutic effect in the skin, but epidermal cells, including keratinocytes, also express this enzyme, indicating the possibility of vitamin D conversion to the active metabolite in the interest area [17].

Even if this information plays an important role in the disease’s cure, it is important to note that the efficacy of the enzyme’s function in psoriasis is still a matter of uncertainty. The current evidence indicates an increase in the proportion of the enzyme’s expression in the dermal mast cells of the lesional skin [18]. Moreover, it is also worth mentioning that the possible mutation/deletion of the enzyme in the skin was associated with the epidermal stratum basale hyperproliferation [19], but the connections with the disease’s particularities are, to our knowledge, lacking.

Therefore we performed a preliminary study whose purpose was to observe the immunohistochemical characteristics of the CYP27B1 enzyme belonging to different severity types of the disease assessed by the Psoriasis Area Severity Index (PASI) score and the Dermatology Life Quality Index (DLQI) score, considering other important immunohistochemical markers such as the cell proliferation antigen Ki67 and the CD45RO+ memory-effector T cells.

## 2. Materials and Methods

### 2.1. Patients’ Selection

Our study received approval from the Ethics Committee of The University of Medicine and Pharmacy of Craiova, Romania, (No. 83/07.04.2023) and was conducted in accordance with the Declaration of Helsinki, with all patients being required to sign informed consent. For the patients younger than 18 years old, agreement was obtained from their legal guardian.

Lesional skin biopsies were taken from 11 psoriasis patients diagnosed at the Dermatology Department of the Clinical Emergency County Hospital of Craiova, Dolj County, Romania, and we divided them into two groups based on the disease severity criteria: Group 1 characterized by patients with mild form of the disease, including 4 patients, and Group 2, the group of patients with a moderate to severe form of the disease, which included 7 patients. The lesional skin sections were taken from the most affected anatomical region of the patients such as the elbow, the knee, lumbosacral area, or the trunk.

The patients’ inclusion criteria were clinical and pathological diagnosis of psoriasis and the absence of use of any treatment (systemic or topical on the involved areas of psoriasis) in the last 4 weeks before the skin biopsy.

The exclusion criteria were topical or systemic use of corticosteroids or other treatments that can affect the immunological response (e.g., antifungal azoles) and diagnosed uremia (as CYP27B1 can be suppressed [20]).

### 2.2. Clinical Data of the Patients

The assessment of the psoriasis severity type was performed considering the EuroGuiDerm guidelines [21]. Therefore, the category of patients with mild psoriasis was described by affected body surface area (BSA) ≤ 10 and PASI ≤ 10 and DLQI ≤ 10, and the one with moderate to severe psoriasis was described by affected BSA > 10 or PASI > 10, and DLQI > 10 [21].

If one patient had a PASI score ≤ 10 but a DLQI score > 10, the disease’s form was considered moderate to severe [22].

Particularities of the clinical context considered to be useful in the immunohistochemical investigations are detailed in Table 1 and Table 2.

### 2.3. Immunohistochemistry, Markers, and Other Variables Measured

Paraffin-embedded sections of lesional skin taken from psoriasis patients under local anesthesia were evaluated in hematoxylin–eosin (HE)-stained slides to confirm the diagnosis and then prepared as follows.

#### 2.3.1. Immunohistochemistry Technique for the CYP27B1 Enzyme

For analyzing CYP27B1 expression, we used the rabbit anti-CYP27B1 antibody (G-5): sc-515903 reagent (Santa Cruz Biotechnology, Dallas, TX, USA) using the immunohistochemistry technique. Formalin-fixed paraffin-embedded 4–5 µm sections were heated in pH buffer using EnVision Flex Target Retrieval Solution (Dako, Agilent Technologies, Santa Clara, CA, USA), followed by blocking of endogenous peroxidase activity using Peroxidase block (Novo Link Kit, Leica Biosystem, Newcastle Upon Tyne, UK). The dilution of the primary antibody was prepared at 1:25 in antibody diluent (Dako, Carpinteria, CA, USA), followed by detection with Max Polymer Detection System (Novo Link Kit, Leica Biosystem, UK), according to the manufacturer’s directions. The antigen–antibody binding was visualized using 3,3′-diaminobenzidine (DAB) (Envision System-HRP Labeled Polymer Anti-Mouse; Dako, Glostrup, Denmark), followed by hematoxylin counterstaining and automated coverslipping (Tissue-Tek Film^®^ Automated Coverslipper-Sakura^TM^, Tokyo, Japan).

For testing the immunohistochemical expression of CYP27B1, we used tissue controls to ensure the accuracy and reliability of our results. The positive control consisted of a tissue sample of kidney known to express CYP27B1 and also healthy skin tissue samples. Both exhibited a positive staining pattern for CYP27B1, confirming the reliability of our immunohistochemical staining method. The negative control involved omitting the primary antibody and was used to help identify any nonspecific staining or background staining that might occur in the absence of CYP27B1 expression.

#### 2.3.2. Immunohistochemistry Technique for the Cell Proliferation Antigen Ki67 and the CD45RO+ Memory-Effector T Cells Marker

Immunohistochemical staining was performed using the BenchMark Ultra IHC/ISH staining system from Ventana Medical Systems, Inc., Marana, AZ, USA, to assess CD45 and Ki67. The monoclonal antibody clones used were LCA, 2B11, and PD7/26 for CD45 and 30-9 for Ki-67.

#### 2.3.3. Immunostaining Assessment

All the slides were tested in a blinded manner by two independent pathologists without knowing the clinical data of the patients.

A scoring system was devised after checking the literature [23,24,25,26], integrating multiple markers while accommodating their unique characteristics within the scoring criteria. Ki67 staining involved estimating the percentage of positively stained nuclei among epithelial cells across high-powered fields. CD45 assessment followed a similar principle, estimating the percentage of positively stained lymphocytes. Meanwhile, CYP27B1 staining intensity was semiquantitatively scored specifically within epithelial cells, independent of cell count or positivity percentage.

CYP27B1 immunohistochemistry was assessed by grading staining intensity on a scale of 0 to 3, with 0 indicating no staining, 1 for weak staining, 2 for moderate staining, and 3 for strong staining. Staining intensity was compared to that of normal skin epidermis, which was scored as strong.

Ki67 was semiquantitatively graded by counting the number of positive cells per ten high-power fields. The median count was then used to categorize the severity into the following grades:

- Grade 0: None, when the median count is less than 10.
- Grade 1: Weak, when the median count ranges from 10 to 30.
- Grade 2: Moderate, when the median count ranges from 31 to 50.
- Grade 3: Strong, when the median count exceeds 50.

Inflammation grades were determined by counting the number of CD45-positive inflammatory cells per ten high-power fields. The grading criteria were as follows:

- Grade 0: No infiltrate when the median count is <10.
- Grade 1: Mild infiltrate when the median count is between 10 and 30.
- Grade 2: Moderate infiltrate when the median count is between 31 and 50.
- Grade 3: Marked infiltrate (severe inflammation) when the median count is >50.

### 2.4. Statistical Analysis

The characteristics of patients were presented as mean with standard deviation (SD) and median with interquartile range (IQR) for continuous variables and as percentages for categorical variables. Nonparametric Mann–Whitney U-test was used to compare continuous variables between the two groups of patients based on the severity of the disease.

We used Chi-square or Fisher’s exact test to assess whether the relationship between the severity of the psoriasis and characteristics of categorical type (the type of psoriasis; Ki67; CYP27B1; levels of enzyme expression in the epidermis) was more than expected by chance. We used GraphPad Prism 10.0.2 for Windows (GraphPad Software, LLC, San Diego, CA, USA) to conduct the statistical analyses. A *p*-value lower than 0.05 was considered significant for all statistical analyses.

## 3. Results

### 3.1. Analysis of the Disease-Related Aspects and Demographic Characteristics

The subjects included in the study (n = 11) were patients of both genders (54.5% male) with mild or moderate to severe psoriasis.

Table 3 summarizes the average demographic characteristics and disease-related aspects, clinical or immunohistochemical, assessed for them.

According to our statistical analysis, considering the demographic factors, there is no significant difference between the analyzed groups.

As expected, the mean values for the PASI and DLQI scores are higher in the moderate to severe group.

The staining assessment for the CYP27B1 enzyme and the immunohistochemical markers was performed as described in the Material and Methods.

Although statistically insignificant, a notable result seems to be present when analyzing the staining score of the CYP27B1 enzyme between the assessed groups (*p* = 0.073). A divergent result is observed for the moderate to severe group as the enzyme seems to have either a staining score of 0 (absent) or 2 (moderate staining), with no intermediary state between.

### 3.2. Results Obtained from Different Severity Types of Psoriasis and Comparison with the Clinical Context

CYP27B1 enzyme expression was analyzed as previously described. For three of our patients, the CYP27B1 enzyme expression was assessed as absent, while the others had weak to moderate staining. None of our psoriasis patients had strong CYP27B1 enzyme staining.

As presented in Table 4, we encountered different Ki67 scores, ranging from weak to strong.

All the analyzed samples were characterized by marked inflammatory infiltrate since the range of CD45RO+ scores varied from 60% to 90%.

**Figure 1:**
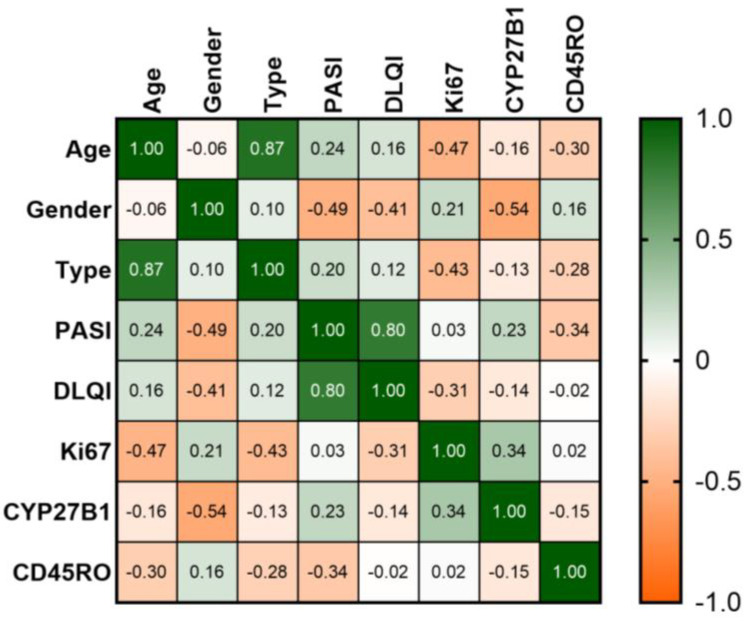
Heatmap of the correlation matrix. Colors range from bright green (strong positive correlation; rho = 1.0) to bright orange (strong negative correlation; rho = −1.0).

As presented in Figure 1, no significant statistical correlation was obtained between CYP27B1 and Ki67 (rho = 0.34, *p* = 0.313) or CD45RO+ (rho = −0.15, *p* = 0.657).

### 3.3. Immunohistochemical Characterization

#### 3.3.1. Healthy Skin and Control

**Figure 2:**
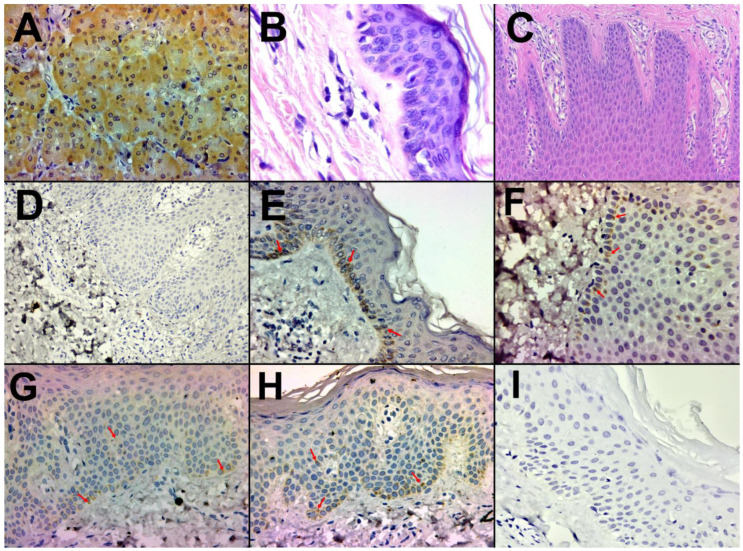
Positive control—Immunohistochemical analysis of paraffin-embedded human kidney tissue labeling CYP27B1, 200× (**A**), HE-stained slides of healthy skin, 200× (**B**), HE-stained slides of psoriasis skin tissue, 100× (**C**). Negative control—Immunohistochemical analysis without the primary antibody of paraffin-embedded healthy skin, 200× (**D**). Positive control—Immunohistochemical analysis of paraffin-embedded healthy skin labeling CYP27B1, score 3, 200×. (**E**). Mild psoriasis—Immunohistochemical analysis of paraffin-embedded human skin tissue labeling CYP27B1 in the epidermal stratum basale, staining score 1, 200× (**F**). Moderate to severe psoriasis—Immunohistochemical staining for CYP27B1—staining score 1 (weak) with ubiquitous distribution throughout the stratum basale and stratum spinosum, 200× (**G**). Moderate to severe psoriasis—Immunohistochemical staining for CYP27B1—staining score 2 (moderate) with ubiquitous distribution throughout the stratum basale and stratum spinosum, 200× (**H**). Moderate to severe psoriasis—Immunohistochemical analysis of paraffin-embedded human skin tissue showing no labeling of the CYP27B1, 200× (**I**). CYP27B1 immunostaining is indicated by arrows.

The immunohistochemical assay for CYP27B1 in kidney tissue showed strong and specific cytoplasmic staining in the renal tubules, as observed in Figure 2A.

Healthy skin samples were evaluated in hematoxylin–eosin (HE)-stained slides (Figure 2B), and positive (Figure 2E) and negative control of the enzyme (Figure 2D) were also performed on them.

The presence of CYP27B1 was observed in a positive manner within the stratum basale of the epidermis in healthy skin samples (Figure 2E). These specific epidermal layers encompass regions where keratinocytes engage in active cellular processes, including the enzymatic conversion of vitamin D into its biologically active form.

#### 3.3.2. Psoriasis Skin

Psoriasis skin samples were evaluated in HE-stained slides to confirm the diagnosis (Figure 2C). No traces of melanin were observed in the analyzed samples involved in the study.

In psoriatic samples, we observed CYP27B1 staining in the stratum basale (Figure 2F) and stratum spinosum of the epidermis (Figure 2G,H). Two cases showed a mild staining (Figure 2G), six cases showed a moderate staining (Figure 2H), and in three cases the enzyme was assessed as absent (Figure 2I).

**Figure 3:**
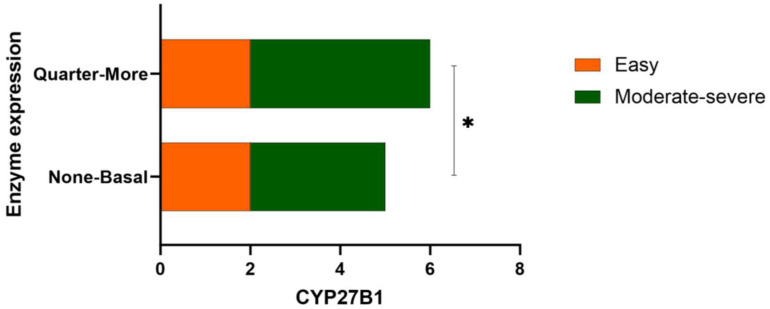
The distribution of the enzyme throughout the epidermal layers. None = enzyme assessed as absent, Basal = distribution of the enzyme in the stratum basale, Quarter–More = distribution of the enzyme throughout at least a quarter or more of the stratum spinosum. * p-value < 0.05.

When analyzing the epidermal distribution of the enzyme regarding psoriasis type of severity, there seems to be some differences between the mild (easy) and moderate to severe groups (*p* = 0.044), as presented in Figure 3.

An interesting result was found for the patients with mild psoriasis assessed by very low PASI scores (PASI = 4.4, respectively, PASI = 5.4) as these patients had a similar distribution of the enzyme to healthy skin, the enzyme being observed in the epidermal stratum basale. However, the registered staining scores were different between the samples of healthy skin (staining score 3) and samples of mild psoriasis skin (staining score 1).

In contrast, for the patients with moderate to severe psoriasis, the enzyme expression seemed to follow an “all or nothing” pattern, with either ubiquitous distribution throughout the stratum spinosum or no expression at all.

In spite of that, the staining CYP27B1 assessment could be significantly correlated with none of the psoriasis scores (*p* = 0.493 for the PASI score and *p* = 0.672 for the DLQI score).

**Figure 4:**
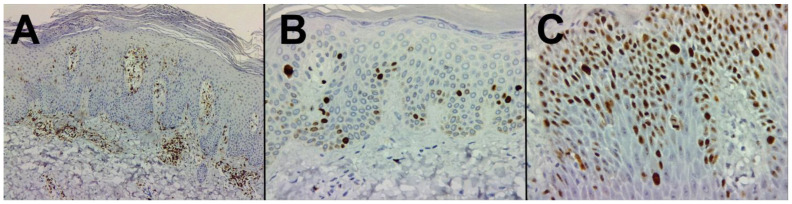
Immunohistochemical analysis of paraffin-embedded human skin tissue labeling CD45RO+ cells, 40× (**A**), Immunohistochemical analysis of paraffin-embedded human skin tissue labeling Ki67, grade 2, 200× (**B**), Immunohistochemical analysis of paraffin-embedded human skin tissue labeling Ki67, grade 3, 200× (**C**).

In psoriatic skin samples, we identified an increase in CD45RO-positive T cells, distributed in the dermal layers of the skin, particularly around blood vessels and within the papillary dermis (Figure 4A).

Immunohistochemical analysis utilizing Ki-67 staining serves as a valuable means to evaluate cellular proliferation. In our examination of psoriatic skin specimens, a discernible augmentation of Ki-67-positive cells was ascertained. Predominantly, the most conspicuous Ki-67 staining was localized within the epidermal strata, notably the stratum basale and suprabasal layers, thereby signifying heightened keratinocyte proliferation in the context of psoriasis. It is noteworthy that the intensity of Ki-67 immunoreactivity exhibited a spectrum of variations amongst distinct psoriatic lesions and among individual patients, wherein certain lesions exhibited a more pronounced Ki-67 staining pattern, effectively mirroring the heterogeneous degree of hyperproliferation within the psoriatic milieu (Figure 4B,C).

## 4. Discussion

The extra-renal activity of the CYP27B1 enzyme became a subject of interest starting in 1981 when high levels of 1,25(OH)_2_ vitamin D were found in the blood of an anephric patient diagnosed with active sarcoidosis [27], the source of bio-activation being later identified in the macrophage which also possesses the ability to express the CYP27B1 enzyme [28].

Since then, the enzyme has proved to be found in a large variety of cells such as keratinocytes, mammary epithelial cells, prostate epithelial cells, enterocytes, pancreatic cells, and immune system cells [29].

Although the gene that encodes the CYP27B1 enzyme is the same for both renal and extra-renal sites—a fact proven by studies performed in patients with known genetic abnormalities of renal enzymes who also manifest reduced production of 1,25(OH)_2_ vitamin D by macrophages [30] and by the findings of the same CYP27B1 messenger RNA in keratinocytes as in the renal cells [31], the factors involved in enzyme regulation seem to be different.

When it comes to the enzyme localized in the kidney, the main regulators for the proximal convoluted tubule are known to be represented by the parathyroid hormone (PTH), fibroblast growth factor (FGF23), and the active metabolite 1,25(OH)_2_ vitamin D, and by calcitonin for the proximal straight tubule [32], while the extra-renal enzyme seems to be locally up-regulated by cytokines, such as interferon-γ (IFN-γ), tumor necrosis factor-α (TNF-α), interleukin (IL)-6, and IL-1β [33].

Regarding the enzyme’s inhibition, both renal and extra-renal enzymes are inhibited by imidazole structures (like ketoconazole and clotrimazole) which bond with the iron in the cytochrome and by naphtoquinones (like menadione) as they interfere with the electron transfer to the enzyme [34].

As mentioned earlier, the most important function of the CYP27B1 enzyme is to bioactivate vitamin D to its active metabolite, 1,25 (OH)_2_ vitamin D. Alongside VDR, this metabolite has a wide variety of actions, some of which are considered classical, including normalizing the function of the parathyroid gland [35], increasing intestinal calcium absorption [36] and renal reabsorption [37], participating in the processes of bone formation, mineralization, and reabsorption [38], and some of which are considered non-classical, including modulating renin and, indirectly, angiotensin II production, playing a role in controlling blood pressure [39], regulating immune processes, both microbial defense and autoimmune events as 1,25(OH)_2_ vitamin D acts as a modulator of the adaptive and innate immune system [40], and promoting differentiation and inhibiting proliferation, with an accumulation of the cells in the beginning of the cell cycle—G_0_/G_1_ [41]. The latter observation might serve as an explanation for the implication of the active metabolite in oncology and skin diseases as well.

The expression of the enzyme in normal skin is increased in the stratum basale, where the keratinocytes are characterized by increased proliferation and is decreased in the following layers where the keratinocytes are marked by increased differentiation [42,43]. This observation supports the attributed role of the active metabolite, 1,25(OH)_2_ vitamin D, that occurs through bio-activation as an inducer of the early stages of the differentiation processes [43,44] and a regulator of the proliferation process [45] as the results obtained in animal studies on genetically modified mice lacking the CYP27B1 enzyme show impaired epidermal differentiation [46].

Conversely, in psoriasis skin lesions, the enzyme has a ubiquitous epidermal expression, being extensively observed throughout the abnormal stratum spinosum [42]; but, to our knowledge, there is no currently evidence of the enzyme’s expression in line with the psoriasis’ severity.

According to our observations, the patients with the mild form of the disease characterized by very low scores of the PASI score (PASI = 4.4, respectively, PASI = 5.4) appeared to have a similar distribution of the CYP27B1 enzyme as detected in healthy skin, whereas the location was only identified in the epidermal stratum basale.

The increased severity type was correlated with the spread of the enzyme in the epidermal stratum spinosum; although, an interesting result seemed to be observed for the patients belonging to the moderate to severe group, as the enzyme was either varyingly spread throughout the stratum spinosum or completely absent. Furthermore, the divergence of the result for the moderate to severe group was also sustained by the staining assessment as the scores obtained were either 0 (absent) or 2 (moderate staining).

The absence of the enzyme in patient’s samples has been associated with poorer prognosis of malignant diseases [26,47]; nonetheless, because of the cross-sectional design of our study, we could not appreciate if the same affirmation is valid for our patients with psoriasis.

Regarding the specific marker of cell proliferation Ki67, the reports from the literature claim that the expression of Ki67-positive cells decreased after a six-week treatment with the vitamin D analog, calcipotriol, topically applied on psoriasis skin, but the effects over dermal inflammation assessed by CD8+ and CD4+ lymphocytes were less conclusive [48].

However, when we analyzed the staining correlation between the enzyme expressed in psoriasis and the Ki67 marker, a significant result could not be obtained.

Contrasting results appear to be registered, instead, in malignant diseases, either of the skin (melanoma) or other territories.

In regard to melanomas, there was an inverse correlation between CYP27B1 expression and Ki67 [26], and similar results were also obtained when analyzing ovarian tumors (the prevalence of positive Ki67 was markedly increased in the area without CYP27B1 expression compared with areas with positive staining of the enzyme) [47], as well as for thyroid cancers [49].

Also, a study performed on malignant diseases assigned the absence or the low expression of the CYP27B1 enzyme with a poorer prognosis of the melanoma [26] and with a decreased overall survival in ovarian cancer when the enzyme was assessed as absent, respectively, or increased aggressiveness when it was assessed as low expression [47].

In our study, three of the patients belonging to Group 2 were characterized by absence of the enzyme in their analyzed lesional skin samples, but due to our preliminary design of the study, we could not offer any conclusion regarding the evolution of the disease, but we highlight the importance of further studies in order to elucidate this direction.

Previous studies have demonstrated the presence of the CYP27B1 enzyme in CD68 (macrophage) surface antigen-positive cells [42], probably because macrophages are one of the cells that express the enzyme [28]. But, in this study, when it came to the lymphocyte inflammatory infiltrate, although cytokines are known to be up-regulators for the enzyme expression, the expression of the CD45RO surface antigen and the enzyme did not seem to be the same, and despite the fact that the scores for the inflammatory infiltrates were high in most of the samples, this did not seem to influence the score of the enzyme’s staining as none of our samples could be given a “very strong” staining score.

Considering the obtained results in the present study, which has proven a potential difference between cases with very mild psoriasis and moderate to severe psoriasis regarding the CYP27B1 enzyme in the epidermal layer, the limitations of this work are represented by the lack of numerous and homogenous study groups, including the lack of more healthy skin samples for a control group, which could reveal whether the results respect a certain statistically significance trend. Other limitations are represented by the lack of other means of investigation such as immunohistochemical quantification of mast cells’ expression of the enzyme, immunochemical markers (VDR, CYP24A1), and serum dosing of vitamin D metabolites, which are issues that will be hopefully further addressed in upcoming studies.

## 5. Conclusions

Extra-renal functions of the CYP27B1 enzyme were studied in a wide category of diseases. But, even if for malignant diseases there was proved to be a connection between the enzyme and the Ki67 marker, in our preliminary study conducted on psoriasis, this does not seem to be the case. Also, the marked inflammatory infiltrate observed in our samples was not associated with the enzyme staining.

Very mild severity assessed by the PASI score was characterized by a uniform enzyme distribution throughout the epidermal stratum basale as observed in healthy skin, and variable distribution into the epidermal stratum spinosum was observed in cases with a more severe type of the disease.

The CYP27B1 enzyme was absent in the sample of three of our patients with moderate to severe psoriasis and we highlight the importance of finding if this could be predictive of a poorer prognosis of the disease.

## Figures and Tables

**Table 1:** Clinical features of patients belonging to Group 1.

Patient’s Number	Gender	Age	PASI Score	DLQI Score
1	Female	62	5.4	9
2	Female	37	4.4	6
3	Male	66	9.6	9
4	Female	17	9.6	6

**Table 2:** Clinical features of patients belonging to Group 2.

Patient’s Number	Gender	Age	PASI Score	DLQI Score
5	Male	59	23.1	21
6	Male	39	44.6	27
7	Male	38	10.2	20
8	Female	76	11.8	17
9	Male	26	11.3	16
10	Female	49	15.0	21
11	Male	65	34.1	11

**Table 3:** Demographic, clinical, and immunohistochemical aspects of the included patients.

Characteristics	Patients (n = 11)	Severity of the Disease		*p*-Value
		Mild (n = 4)	Moderate-sever (n = 7)	
Age				0.705
mean ± SD	48.55 ± 18.6	45.5 ± 22.93	50.29 ± 17.43	
median (IQR)	49 (37–65)	49.5 (22–65)	49 (38–65)	
Gender, Male (n, %)	6 (54.5%)	1 (25%)	5 (71.4%)	0.242
PASI				0.008 ***
mean ± SD	16.28 ± 11.3	7.25 ± 2.74	21.44 ± 13.31	
median (IQR)	11.3 (9.6–23.1)	7.5 (4.65–9.6)	15.0 (11.3–34.1)	
DLQI				0.008 ***
mean ± SD	14.82 ± 7.04	7.5 ± 1.73	19.0 ± 5.0	
median (IQR)	11.3 (9.6–23.1)	7.5 (6.0–9.0)	20.0 (16.0–21.0)	
Ki67				0.757
1 (n, %)	4 (36.4%)	1 (25%)	3 (42.9%)	
2 (n, %)	3 (27.3%)	1 (25%)	2 (28.6%)	
3 (n, %)	4 (36.4%)	2 (50%)	2 (28.6%)	
CD45RO+				0.214
mean ± SD	81.82 ± 10.79	87.5 ± 5	78.57 ± 12.15	
median (IQR)	90 (70–90)	90 (82.5–90)	80 (70–90)	
CYP27B1				0.073
0 (n, %)	3 (27.3%)	0	3 (42.9%)	
1 (n, %)	2 (18.2%)	2 (50%)	0	
2 (n, %)	6 (54.5%)	2 (50%)	4 (57.1%)	

*** *p*-value < 0.01.

**Table 4:** Staining assessment of the enzyme and analyzed markers.

Patient’s Number	Psoriasis Severity Type	CYP27B1 Staining Score	Ki67 Staining Score	CD45RO+ Staining Score
1	Mild	1	2	90%
2	Mild	1	3	80%
3	Mild	2	1	90%
4	Mild	2	3	90%
5	Moderate to severe	2	1	70%
6	Moderate to severe	2	3	90%
7	Moderate to severe	0	1	90%
8	Moderate to severe	2	2	80%
9	Moderate to severe	2	3	60%
10	Moderate to severe	0	2	90%
11	Moderate to severe	0	1	70%

## Data Availability

The data presented in this study are available on request from the corresponding author. The data are not publicly available due to patients’ privacy rights.
